# Communities on the move: community participation in health in rural territories of Buenaventura District in Colombia

**DOI:** 10.1186/s12939-020-01239-8

**Published:** 2020-10-26

**Authors:** Laura Catalina Blandón-Lotero, Marta Cecilia Jaramillo-Mejía

**Affiliations:** 1 Young Researcher Minciencias, Department of Organizational Management, Faculty of Economic and Administrative Sciences, Universidad icesi., Cali, Colombia; 2 Full-Time Professor, Department of Organizational Management, Faculty of Economic and Administrative Sciences, Universidad Icesi., Cali, Colombia

**Keywords:** Community participation, Health, Colombian Pacific littoral, Qualitative comparative analysis (QCA), Primary health care, Disperse rural area, Colombia, participación comunitaria, salud, Litoral Pacífico colombiano, Qualitative Comparative Analysis (QCA), Derechos sociales, Comunidad rural, Atención Primaria en Salud, Marginalidad, inequidad, área rural dispersa, Colombia

## Abstract

**Background:**

Social and community participation is a fundamental component of the development of renewed primary healthcare (PHC). With the recognition of health as a right, such participation is a significant part of the design of public policies aimed at this sector. These policies contribute not only to overcoming inequity in the provision of this type of services but also to a reduction in social inequalities as a whole. Through a comparative analysis, this study aimed to explain the conditions through which ethnic-rural territories of the Colombian Pacific coast participate in health to contribute to the generation of policies and programs in territories with similar conditions.

**Methods:**

The work was developed through the use of multiple techniques and strategies for information collection and analysis. These include several semi-structured interviews, multiple observation exercises and analysis based on a set theory, i.e., qualitative comparative analysis (QCA). The latter aims to develop a model that provides a count of the main causal combinations that allow high community participation in health.

**Results:**

Key findings include how the trajectory of social mobilization and existence of a robust community social fabric became two critical conditions for community participation in the context of social exclusion. The presence of variables such as the implementation of PHC, guarantee of social rights, and trust in institutions, is underestimated as sufficient causal conditions for obtaining this result. Therefore, it is essential to recognize the existence, validity, and importance of processes, experiences, and resourcefulness of political natures, which aim at transforming the daily reality of the inhabitants of these communities. These also set a potential space and scenario for managing the communities’ main problems, including health, in the absence of institutionality that guarantees access to their social rights.

**Conclusion:**

This study points out the importance of understanding community participation as a political activity, expanding exchange dynamics and dialogs between institutions, rulers, and communities to provide social responses in health and well-being to communities and to understand local realities and their own community dynamics.

## Resumen

**Introducción:**

La participación social y comunitaria se destaca como un componente fundamental para el desarrollo de la Atención Primaria en Salud (APS) renovada. Así, a través del reconocimiento de la salud como un derecho, dicha participación forma parte esencial del diseño de políticas públicas orientadas a ese sector. Estas no solo contribuyen a la superación de la inequidad en la oferta de servicios de este tipo, sino también a la reducción de desigualdades sociales en su conjunto.

**Objetivo:**

Explicar las condiciones, a través de las cuales un grupo de territorios étnico-rurales de la costa del Pacífico colombiano participa en la salud, con el fin de contribuir a la generación de políticas y programas en territorios con las mismas características.

**Métodos:**

El trabajo se desarrolló mediante la utilización de múltiples técnicas y estrategias de recolección y análisis de información. Entre ellas pueden destacarse, desde la realización de un significativo número de entrevistas semiestructuradas y múltiples ejercicios de observación participante, hasta la utilización de análisis, basados en teoría de conjuntos, como es el caso de “Qualitative Comparative Analysis” (QCA). Este último desarrolla un modelo, que da cuenta de las principales combinaciones causales que consienten, a través de su interacción, una alta participación comunitaria en salud.

**Resultados:**

Entre los principales hallazgos puede señalarse cómo las trayectorias de movilización social y la existencia de un tejido social comunitario robusto son dos condiciones importantes para la existencia de participación comunitaria en contextos de exclusión social. Además, se subestima la presencia de variables como: la implementación de la APS, la garantía de derechos sociales, y la confianza en las instituciones, como condiciones causales suficientes para la obtención del resultado. Desde allí, debe reconocerse la existencia, vigencia e importancia de procesos, experiencias y potencialidades de orden político, orientadas a transformar la realidad cotidiana por parte de los habitantes de estas comunidades, como un potencial espacio y escenario de gestión de las principales problemáticas de las comunidades, entre ellas la salud, frente a la ausencia de institucionalidad, que garantice el acceso a sus derechos sociales.

**Conclusión:**

Se señala la importancia de entender la participación comunitaria como una actividad política de dinámicas de intercambio y diálogos entre las instituciones, los gobernantes y las comunidades para dar respuestas sociales en salud y bienestar a las comunidades, desde la comprensión de las realidades locales y las dinámicas comunitarias propias.

## Introduction

Social and community participation is a fundamental component of the development of renewed primary healthcare (PHC). With the recognition of health as a right, such participation is a significant part of the design of public policies aimed at this sector. These public policies contribute to overcoming inequity, provided healthcare service, and to the reduction in social inequalities [1]. The concept of community participation has evolved from the Alma Ata Declaration in 1978 through the Astana Declaration in 2018. We assume the concept of community participation as the process of collective deliberation that contributes to the empowerment of communities and governance of the healthcare system to ensure the right to healthcare access [2, 3].

Studies have identified variables associated with community participation in health as processes of empowerment. These variables are individual intrinsic motivations, community-level trust, strong external links, institutional support processes, social capital, leadership, community resources, agency characteristics, community engagement, and organizational structure [4–10]. However, it is necessary to offer new perspectives to strengthen long-term community capacities, based on empirical evidence, on the characteristics of community participation when presence of the state is weak or absent, and inequalities and social exclusion are ingrained [9, 11].

In 2018, Colombia had 48,258,494 inhabitants, with 3.9% indigenous [12] and 9.3% Afro-Colombians [13]. The largest population of African descent (25.5%) is located in the Pacific Littoral [12]. The population of the Pacific coast lives in precarious social conditions and has a low educational level, without access to healthcare services and leisure activities. This population inhabits areas with elevated levels of armed conflict, amid illegal groups, and drug trafficking routes. Although the Afro and indigenous populations are considered a minority, Colombia is rich in ethnic and cultural diversity.

Based on the study “Viability to Develop a Model Based on Primary Health Care” (Colciencias 2015), it was possible to establish the critical panorama of health inequities in the territories of the Colombian Pacific Littoral. The population is dispersed across a vast rural area, is geographically isolated with weak institutions and has the most critical social indicators in the country (health, poverty, and inequity), with a multidimensional poverty index of 37.6%. Rural areas in Colombia have limited access to healthcare services, given the inability of the Colombian health authorities to adapt service delivery models and the lack of information systems to evaluate local health plans. Jaramillo-Mejía et al. found evidence on the presence of participation processes and community resources on self-health (i.e., ancestral health practices) in different levels of development within community participation processes, despite flawed healthcare services and the absence of state institutions. While the identification of community participation variables has yielded noteworthy results, few studies have assessed them in sociopolitical contexts, such as those found in ethnic communities in the Colombian Pacific. Little attention has been paid to how community participation occurs within highly dispersed rural areas and what variables lead to and shape participatory processes in state-absence contexts [14].

Based on the lack of information about community participation in health, we established these questions: What is behind the processes of participation? How could it be explained that some territories have more organized processes of community participation in health than others? The study aimed to explain the causal conditions and interactions that occur in community participation processes in the health of ethnic-rural territories exposed to conditions of inequality and exclusion of the Colombian Pacific Coast of Buenaventura District.

## Background

### Colombian healthcare system

The current healthcare system in Colombia is a public service with private and public providers. It began in 1993 with the implementation of Law 100 and the goal of achieving universal coverage from 17.4% in 1990 to 9.6% in 2018 [15]. This healthcare system is based on the insurance of the population through a health promoter institution (EPS – Empresa Promotora de Salud) that provides healthcare services through public and private entities called health provider institutions (IPS – Institución Prestadora de Salud). In 1990, > 80% of IPS were public. Presently, > 75% of IPS are private. Small municipalities located in the Pacific Coast of Colombia have low coverage of healthcare services, since the IPS are located in the main cities, far from rural and scattered areas (i.e. the Buenaventura District has on only one public hospital in Puerto Merizalde called ESE San Agustin [ESE-Empresa Social del Estado]) [16].

## Methods

### Study setting

**Fig. 1:**
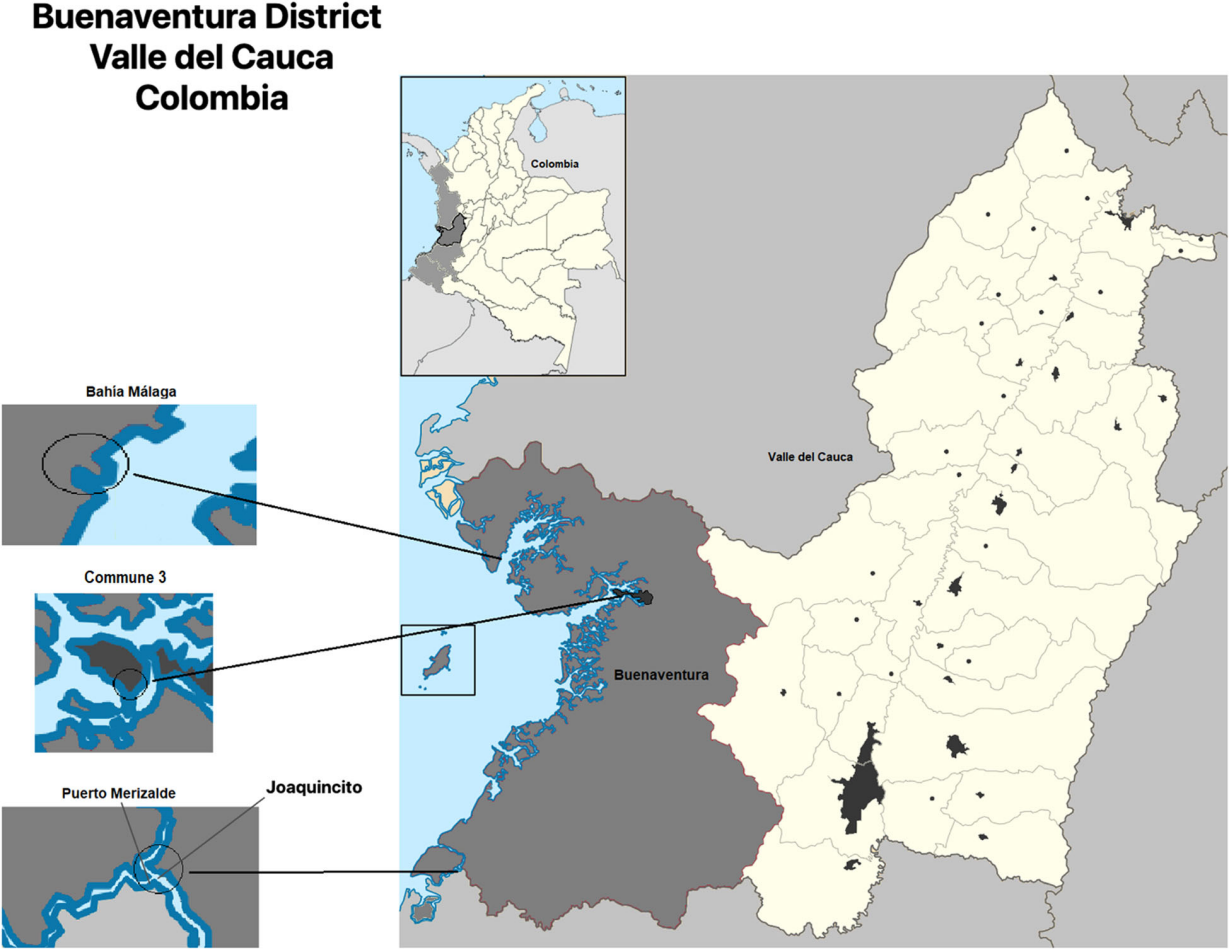
Localization of the Buenaventura District, Valle del Cauca, Colombia. Source: Adapted and edited for our work by the authors. From Wikimedia Commons, free access (https://commons.wikimedia.org/wiki/File:Colombia_-_Valle_del_Cauca_-_Buenaventura.svg)

This study was conducted in the Buenaventura District in Colombia, a tropical country in South America, and the only port in the Colombian Pacific located in the west of the Valle del Cauca Department with an extension of 6078 km^2^. We studied six territories in the Buenaventura District, which comprise the afro communities of the Commune 3, La Sierpe, La Plata, Miramar, Puerto Merizalde, and the indigenous community Joaquincito. The Commune 3 is in the urban area of Buenaventura. To the north of the city, Bahía Málaga (Malaga Bay), are the sidewalks of La Sierpe, La Plata, Mangaña, and Miramar, to the south, along the River Naya, are Puerto Merizalde and the Eperara Siapidara Shelter in Joaquincito. The Commune 3 was populated as a result of internal migration, and the five rural territories require river and maritime transport of 2 or 3 h to the city of Buenaventura (Fig. 1) [17].

### Theoretical framework

In this study, we adapted the conditions identified by Johnson and Sofaer’s model, Goodman’s work in racial and ethnic communities, and Jackson’s study of the socioenvironmental conditions that make participation possible or limit it [18–22]. We identified three categories associated with community participation in health and empowerment: sense of community, contextual causal condition, and historical causal condition. The subcomponents of these categories can be adapted according to the sociopolitical context of the investigated territories [9].

The category sense of community is based on Jackson’s study that includes factors of community action, such as presence of a positive social environment and ability to work together, link to one another, and participate. Jackson states that “community capacity relates to action accruing from collective action rather than being an aggregate of individual abilities [23].” The second category, contextual causal condition, is based on Johnson and Sofaer’s model regarding the characteristics of community health coalitions. These authors found that confidence, adaptation, and dedicated staff contribute to the sustainability of these coalitions [9]. Lastly, Goodman et al. identified the meaning, history, values, and power of a community as the main conditions that determine community, setting the theoretical framework for the third category historical causal condition [24, 25].

### Qualitative comparative analysis

Qualitative Comparative Analysis (QCA) is based on a set theory to examine the causal relationships between conditions that, usually combined, are considered as necessary or sufficient for the production of a particular outcome. Unlike statistics, a set theory does not attempt to identify the model with the best possible fit but the diversity of solutions that explain the outcome. QCA focuses on the comparative logic of classical qualitative studies but with a greater degree of formalization and complexity [18]. We identified QCA and crisp set (csQCA) as the method and technique that captures the complexity and heterogenic causal relationships and evaluates the specific conditions of the context of community participation processes [18, 26]. We applied this technique through a process comprising three basic moments: 1) case selection and description, 2) QCA proposal (“analytic moment”), and 3) interpretation.

### Study sample

We identified and selected community leaders and representatives of ethnic organizations in the selected territories of the Colombian Pacific coast that include the community councils of Afro-Colombian communities and indigenous shelter of Eperara Siapidara. We selected samples for the questionnaire, focus groups, and in-depth interview. For the questionnaire, we obtained participation of 90 community leaders, 15 for each territory, except in Sierpe, where we only achieved participation of six individuals, therefore excluding them from the study. We applied the stakeholders’ instrument to focus groups of ten key individuals per group, comprising leaders and members of the board of directors of the community councils, and the governor and health promoter of the indigenous shelter.

### Defining the conditions and outcome

The process of defining the conditions and outcome involved the analysis of models for the development of community health capacities, theoretical frameworks, review of the literature on participation, and debate and refinement of the conditions based on fieldwork. In the selection of conditions of the study, we followed the ease of capturing the presence or absence of that condition and its relevance in the theoretical review [27].

**Fig. 2:**
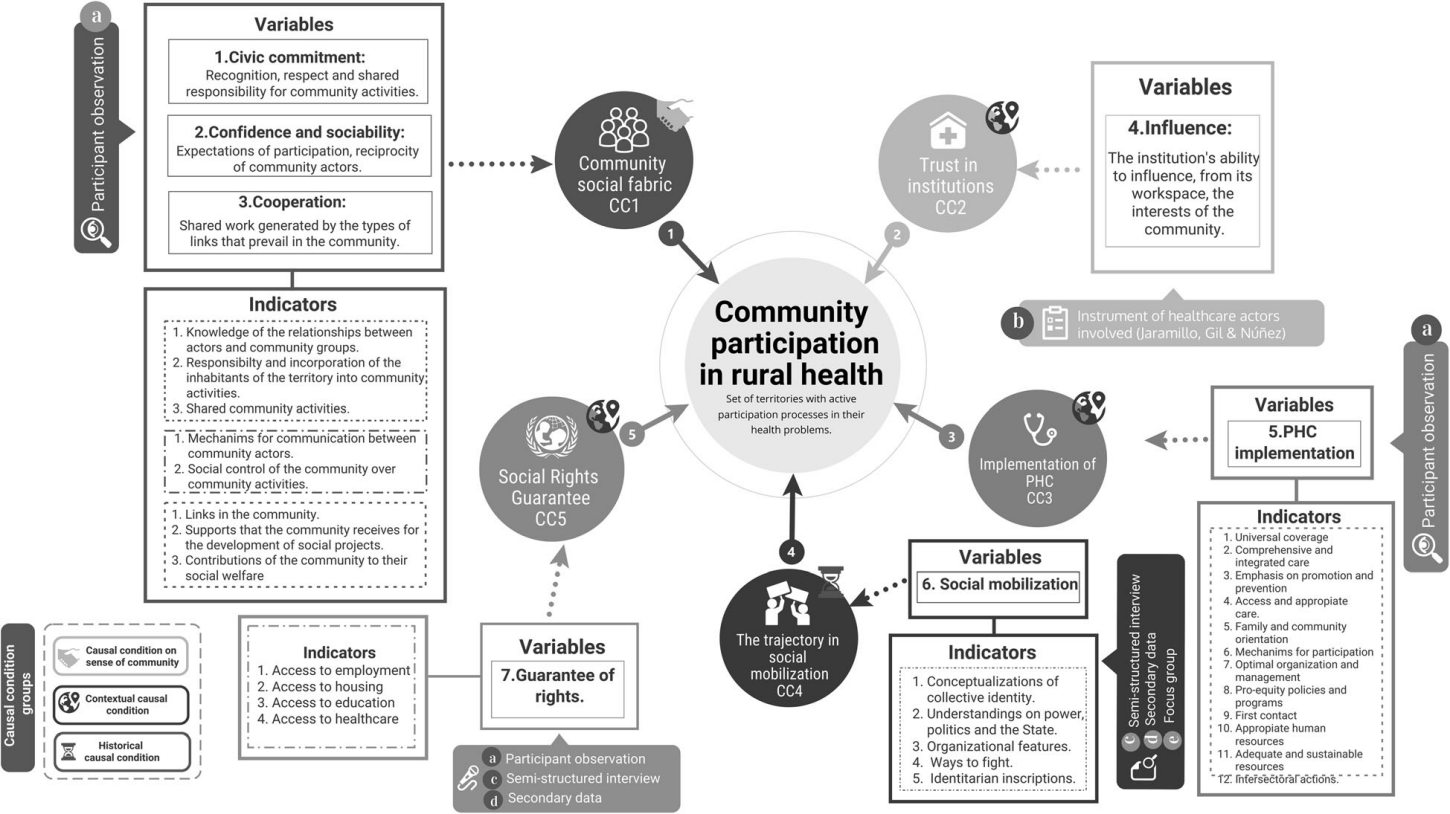
Causal analytical model for community participation in health in the Colombian Pacific Littoral. Source: Own elaboration (Jaramillo-Mejía MC, Blandón-Lotero LC. 2020)

We identified five causal conditions that fit into the three categories adapted from Jackson, Johnson and Sofaer, and Goodman’s studies [9]. Figure 2 presents a more detailed description of the causal conditions related to the outcome of interest (community participation in rural health) and associated variables and indicators.

### Data collection

Between December 2016 and March 2018, we collected primary and secondary qualitative and quantitative data using mixed methods approach (labeled [a] through [e] in Fig. 2), that we later organized into five datasets representing each causal condition (labeled [1] through [5] in Fig. 2). For the dataset for CC4 and CC5 we used secondary data [d] about social mobilization processes and reports from the National Administrative Department of Statistics on the guarantee of rights (employment, housing, education, and health) in the Colombian Pacific (Variable 7, Fig. 2) [28].

### Scoring the cases

We developed an array of data to simplify the information of each case and assigned values to the result of interest and five causal conditions in the six territories to construct a dichotomic data matrix. CsQCA assigns values from zero [0] when each criterion is not present and one [1] when each criterion is present. Table 1 shows the set of criteria for each causal condition. For the result and majority of the causal conditions, we defined a variable chained to a series of attributes and indicators in a way that would allow us to evaluate multiple dimensions in each concept (Fig. 2). For example, in the fourth causal condition (CC4) “mobilization,” we defined the indicators that could be used to establish the cutoff line between communities that do or do not have a mobilization trajectory.

**Table 1:** Description of conditions and outcome used in community participation in rural health and criteria for assigning value 1

Conditions and outcome	Description	Criteria for assigning value 1
**Sense of community**		
Community social fabric (CC1)	•Community, family and social networks and conditions for the relationship with a sense of community.•Solidary support within the inhabitants.	• Strong civic commitment (presence of more than 1 indicator)• High confidence and sociability (presence of both indicators)• Strong cooperation (presence of both indicators)
**Contextual causal condition**		
Trust in institutions (CC2)	• The institution’s ability to influence, from its workspace, the interests of the community^a^. ^a^Influence is understood as an antecedent or proof of trust placed in institutions [29].	• Medium or high influence degree of influence of institutional actors in the social fabric.
Implementation of PHC (CC3)	• Presence of elements of the renewed PHC, defined by the Pan American Health Organization (PAHO).	• Presence of 6 or more elements of the renewed PHC.
Social Rights Guarantee (CC5)	• Territories with access to social right	• Presence of at least three of the four indicators: employment, housing, education, healthcare.
**Historical causal condition**		
The trajectory social mobilization (CC4)	•Territories that have historically participated in defense of the collective territory, political participation, and their social rights through a defined organizational agenda.	• Presence of conceptualizations of collective identity by ethnic organizations.• Understandings about power, politics, and the state by community leaders.• The leaders identify the organizational characteristics of the territory.• Community leaders identify social mobilization as a form of struggle. • Identification of collective identity construction processes by community leaders.
**Outcome**		
Community participation	•Set of territories with active participation processes in their health problems.	•Presence of organizations working for common interests. •Participation of organizations in decision-making spaces. •Perceived presence of community leadership. •Planning and taking actions to improve the health of the territory. •Identification and prioritization of health needs and problems by the community.

^a^1, presence of the condition; 0, absence of the condition

Source: Elaboration based in Jaramillo-Mejía, Gil, and Núñez [30]; Mira and Rojas [31]; Parra [32]; Nakamura and Siregar [33]; Téllez [34]; Goodman [25] and Jackson, Cleverly, Poland, Burman, Edwards, and Robertson [35]

### CsQCA analysis

The dataset of case scores was analyzed using fsQCA software to evaluate the combinations of conditions that led to the outcome of strong community participation in health. Based on the allocation of these thresholds, we obtained a binary data array.

Using the obtained truth table (occurrence and non-occurrence of the result [Annexes 1 and 2]), we eliminated the configurations that were not present in the data collection, which were the cases that did not have empirical evidence (logical remnants). The analysis of the truth table was based on consistency and coverage. Consistency indicates the percentage of the cases within a configuration of conditions that show the result of interest. Coverage indicates the proportion of cases that have the condition and result of interest between all cases that show that condition. We verified the contradictory cases using the TOSMANA software (a tool for small-n analysis, QCA) software, using Venn diagrams (Annex 3).

We conducted a necessity analysis showing that, for the occurrence of community participation in health (Annex 4), there is only one necessary condition (consistency, 1.000000, and coverage, 1.00000). This is the trajectory in social mobilization, which is always present when the result of interest occurs. Subsequently, the calibrated information was processed using a minimization algorithm, obtaining three types of solutions: complex, intermediate, and parsimonious. Here, we opted for complex and intermediate solutions since we made conservative use of counterfactuals, indicating that we had empirically observable results [36].

The interpretation of the results involved the revision of the causal configurations obtained by complex and intermediate solutions and analysis of the truth table. From that revision, we conducted two processes: we returned to the cases and information collected in the field and evaluated the theory to identify causal mechanisms or forces that existed between the configuration and processes of community participation on the Colombian Pacific coast.

## Results

Community participation in health was present in four territories (Commune 3, Puerto Merizalde, Joaquincito, and La Plata) and absent in two territories (Mangaña and Miramar). Table 2 shows the values of each causal condition in the six case studies, with the presence or absence of the outcome.

**Table 2:** Synthetic matrix of calibrated data

Case	Sense of community	Context			History	Outcome
	Fabric	Institutions	PHC	Rights	Mobilization	Community participation
**Commune 3**	1	0	1	1	1	1
**Puerto Merizalde**	0	1	1	0	1	1
**Joaquincito**	1	0	0	0	1	1
**La Plata**	1	0	0	0	1	1
**Mangaña**	0	0	0	0	0	0
**Miramar**	1	0	0	0	0	0

Source: Own elaboration

Of the four cases with active processes of community participation, 100% had a history of social mobilization, at least three of them had a strong community social fabric, and of the two cases without community participation, only one has a strong social fabric. Moreover, in two cases with active community participation processes, at least two conditions related to the context of the territory were present, but they only share one condition: implementation of the PHC strategy. In territories where there is no community participation, there is also neither contextual causal condition nor trajectory in social mobilization.

Table 3 shows the results of complex and intermediate solutions, coverage level, and consistency of cases with active processes of community participation in health.

**Table 3:** Complex and intermediate solution results

Solutions	Consistency	Coverage	No. of cases
fabric*mobilization	1.000000	0.500000	2
*institutions*phc*mobilizacion	1.000000	0.250000	1
fabric*phc*mobilization*rights	1.000000	0.250000	1
Solution coverage:	1.000000		
Solution consistency	1.000000		

Source: Authors. Calculations performed using fsQCA 2.5 software

Based on the causal condition model (Fig. 2), we described three complex and intermediate solutions to identify patterns in the community participation processes in health that help explain the interactions between causal conditions that lead to the outcome.

### Pathway 1

#### Interactions between the causal condition on a sense of community and historical causal condition

The combination of a strong community social fabric and presence of social mobilization trajectories showed, as a result, the presence of active processes of community participation in health, despite the absence of all contextual causal conditions. The cases in the indigenous shelter of Joaquincito and village of La Plata (Bahía Málaga), have shown the presence of community participation and strong presence of community networks, family, and social and favorable relationship conditions, including a sense of solidarity and support, protection of their territories, political participation, and inclusion of social rights through an organizational agenda. The pathways of social mobilization of these territories were configured through the defense of natural resources and each community’s own model of development, within which they include the health model.

### Pathway 2

#### Interactions between contextual causal condition and historical causal condition

The combination of trust in institutions, implementation of the PHC strategy, and presence of trajectories in social mobilization, in addition to the absence of causal conditions of sense of community and guarantee of rights, also leads to the presence of active processes of community participation. The village of Puerto Merizalde is the only case that is covered by this solution. Contrary to the three other cases, this was the only one that did not include the presence of a strong community social fabric. The presence of the causal condition “trust in institutions” is explained by how the community interprets the presence of a first-level hospital in a rural context without state institutions. Concerning the historical causal condition, the processes of social mobilization in Puerto Merizalde, besides being configured through the defense of the collective territory, has been marked by peace-building processes and peaceful resistance of the Naya River communities, in territories profoundly affected by actors outside the law and with high rates of human rights violations.

### Pathway 3

#### Interactions among the contextual causal condition, sense of community causal condition, and historical causal condition

The combination of a strong community social fabric, implementation of the PHC strategy, presence of trajectories in social mobilization, minimum guarantee of social rights, and absence of trust in institutions is the pathway that leads to active processes of community participation in health. This solution refers to the case in Commune 3 in Buenaventura District, the only case in which the outcome has a minimum guarantee of social rights and shows the interaction between the causal condition groups.

However, according to the results of the parsimonious solution, the most relevant causal condition seems to be the trajectory in social mobilization, to which the role of necessity is credited. As shown in Table 4 (complex and intermediate solutions), this causal condition is present in all cases where the result of interest is present: Commune 3, Puerto Merizalde, Joaquincito, and La Plata (Bahía Málaga).

**Table 4:** Results with a parsimonious solution

Solutions	Consistency	Coverage	No. of cases
fabric*mobilization	1.000000	0.500000	2
*institutions*phc*mobilizacion	1.000000	0.250000	1
fabric*phc*mobilization*rights	1.000000	0.250000	1
Solution coverage:	1.000000		
Solution consistency	1.000000		

Source: Calculations performed using fsQCA 2.5 software

## Discussion

In different ethnic-rural territories of the Buenaventura District, the history and power of communities were important elements in the creation of forms of community participation in health. In this way, four of six study territories generated participatory health processes aimed at addressing health inequities through processes involving the collective identity of basic movements; understanding of the relationship with power, politics, and state; organizational characteristics; forms of struggle; and identity inscriptions of communities. These findings are in line with those that assert the importance of organizational structure and community leadership in the processes of empowerment and participation in health, exerting pressure across communities on health policymakers that address health inequities [9, 23, 37].

Chilaka states that many of these actions conducted by community organizations and leaders do not impact decision-making at a national level by being immersed in entrenched power structures [38], while authors such as Strasser et al. emphasize the importance of community participation in the decision making process [39]. Our findings are in line with Strasser’s and with the literature where territories have demonstrated the transformative potential of iterative cycles of collective action [11, 39], and where community empowerment is closely associated with social movements [40].

The people of the Buenaventura District and ethnic-rural areas that make up this study, led a civic strike as a mechanism of enforceability of social, economic, and cultural rights in May 2017. The statement of petitions made to the government as a result of the strike included eight points, with the first being “coverage in prevention and care in low, medium, and high complexity health and traditional medicine.” However, short-term gains in achieving a dialog table and “agreement” with the government for the enforceability and guarantee of their rights did not necessarily translate into structural changes in the healthcare system. This situation is in line with Cassetti et al.’s understanding of community participation as the process to engage the communities in the decision-making, using methods of collaboration and empowerment; however, the response of the government is imminent for the guarantee of rights and their real involvement in the decision making [41].

There are three sufficient causal configurations in the generation of participatory processes in the ethnic-rural areas of Buenaventura District. The first emphasizes the importance of a sense of community through the causal condition “community social fabric,” i.e., high civic engagement, cooperation, trust, and sociability. Moreover, the presence of the causal condition “trajectory in social mobilization” is necessary, underestimating the presence of context conditions, such as trust in institutions, implementation of the PHC strategy, and guarantee of social rights. The absence of the first two conditions goes against Johnson and Sofaer’s model given the importance to have trust in institutions and excellence of health workers to create higher level community participation in health [9].

Returning to the empirical knowledge of the cases of indigenous shelter of Joaquincito and sidewalk of La Plata (Bahía Málaga), it is important to note that the participatory processes of these two territories relate to a significant form of social capital. Both are characterized by highly organized communities, with a broad sense of collective, solidarity-driven economies, and concern for the preservation of their community resources in health, i.e., midwives, traditional medicine, herbalists, “pildeceros,” and bonesetters. The community concept of these two territories is manifested organizationally and politically in spaces for participation of each ethnic group (assemblies) to plan and solve main social issues, including health problems.

The second causal configuration indicates that, in addition to the trajectory in social mobilization, participatory processes are associated with trust in institutions and implementation of the PHC strategy. The case data contained in this causal configuration suggest that there may be a combination of participation driven by the formal mechanisms of health institutions (understood as collaboration) and basic processes of communities but does not necessarily translate into a broader health participation process. This result is particularly interesting since it is contradicted by authors like Scott who associate health participation with strong external links with the community and institutional support processes [4].

A final causal configuration, in addition to relating participation processes to the sense of community and implementation of the PHC strategy, involves the guarantee of minimum social rights. According to the results obtained in this study this contextual causal condition is an irrelevant. In contrast, the absence of this condition in the three other cases seem to support studies that have shown that, in the face of contexts of exclusion and marginality, communities mobilize to demand their rights [42–44].

## Conclusions

The study recognized three [3] sufficient causal configurations in the generation of community participation processes in ethnic-rural areas of the Buenaventura District. The first configuration, the causal condition “community social fabric” and “path in social mobilization,” highlights the importance of a sense of community.

The participatory processes, in the Joaquincito indigenous shelter and Puerto Merizalde (Afro-Colombian), are related to a significant form of social capital. These two territories have organized communities, with a broad sense of collective, solidarity-driven economy, and concern for the preservation of their health community resources, i.e., midwives, traditional medicine, herbalists, “pildeceros,” and medicine men. The community concept is that territories are manifested organizationally and politically in the areas of internal participation of each ethnic group (assemblies), where mechanisms are proposed to plan and solve their main social and healthcare problems.

Thus, this study identified conditions through which ethnic-rural territories of the Colombian Pacific Littoral, i.e., those involved in health in the context of social exclusion, can contribute to the dissemination of information and case studies and generation of health policies and programs by territorial authorities.

The causal conditions and configurations identified in this study summarize the importance of understanding participation in health as a political activity and highlight the need to expand exchange dynamics and dialogs among institutions, rulers, and communities to provide social responses in health and well-being to communities from understanding local realities and community dynamics.

## Data Availability

The datasets used and/or analyzed during the current study are available from the corresponding author on reasonable request.

## Abbreviations

AUC: Autodefensas Unidas de Colombia
CC: Causal condition
CP: Community participation
DANE: Departamento administrativo nacional de estadística
ELN: Ejército de liberación nacional
ESE: Empresa social del estado
FARC: Fuerzas alternativas revolucionarias de Colombia
FUNDESCODES: Fundación espacios de convivencia y desarrollo
MPI: Multidimensional poverty index
NICE: National Institute for health and care excellence
PAHO: Pan American health organization
PHC: Primary health care
QCA: Qualitative comparative analysis
SENA: Servicio nacional de aprendizaje
TOSMANA: Tool for small-N analysis
WHO: World health organization
